# Supplementary material for: Shuttle peptide delivers base editor RNPs to rhesus monkey airway epithelial cells in vivo
Source: Nat Commun. 2023 Dec 5;14:8051. doi: 10.1038/s41467-023-43904-w (PMC10698009; doi:10.1038/s41467-023-43904-w)
Supplement: Supplementary file 1 — Supplementary Information [file 41467_2023_43904_MOESM1_ESM.pdf]

Supplementary Information

Shuttle Peptide Delivers Base Editor RNPs to Rhesus Monkey Airway Epithelial Cells *In Vivo*

Katarina Kulhankova[1]; Soumba Traore[1]; Xue Cheng[2]; Hadrien Benk-Fortin[2]; Stéphanie Hallée[2]; Mario Harvey[2]; Joannie Roberge[2]; Frédéric Couture[3]; Sajeev Kohli[4,5,6]; Thomas J. Gross[7]; David K. Meyerholz[8]; Garrett R. Rettig[9]; Bernice Thommandru[9]; Gavin Kurgan[9]; Christine Wohlford-Lenane[1]; Dennis J. Hartigan-O'Connor[10,11]; Bradley P. Yates[14]; Gregory A. Newby[4,5,6,14]; David R. Liu[4,5,6]; Alice F. Tarantal[11,12,13]; David Guay[2]; Paul B. McCray, Jr.[1]*

[1]Department of Pediatrics, University of Iowa, Iowa City, IA, USA; [2]Feldan Therapeutics, Quebec, Canada; [3]TransBIOTech, Lévis, Quebec, Canada; [4]Merkin Institute of Transformative Technologies in Healthcare, Broad Institute of MIT and Harvard, Cambridge, MA, USA; [5]Department of Chemistry and Chemical Biology, Harvard University, Cambridge, MA, USA; [6]Howard Hughes Medical Institute, Harvard University, Cambridge, MA, USA; [7]Department of Internal Medicine, University of Iowa, Iowa City, IA, USA; [8]Department of Pathology, University of Iowa, Iowa City, IA, USA; [9]Integrated DNA Technologies, Coralville, IA, USA; [10]Department of Medical Microbiology and Immunology, School of Medicine, UC Davis, Davis, CA, USA; [11]California National Primate Research Center, UC Davis, Davis, CA, USA; [12]Departments of Pediatrics and [13]Cell Biology and Human Anatomy, School of Medicine, UC Davis, Davis, CA, USA; [14]Department of Genetic Medicine, Johns Hopkins University School of Medicine, Baltimore, MD, USA

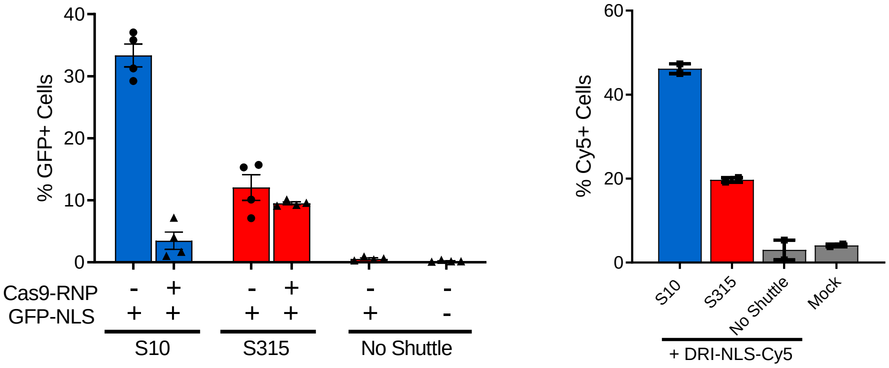

Supplementary Figure 1. a) Effect of additional anionic charge density from Cas9 RNP on GFP delivery to CFF-16HBEge cells with indicated shuttle peptides. Primary data for Fig. 1c. Each closed circle or triangle represents a technical replicate. Results plotted as mean ± SEM. **** denotes $P<0.0001$; ns: non-significant by two-tailed unpaired t-test (brackets). **b)** Delivery efficiency of DRI-NLS-Cy5 to CFF-16HBEge cells with indicated shuttle peptides. Each closed circle or triangle represents a technical replicate. Results plotted as mean ± SEM. Source data are provided as a Source Data File.

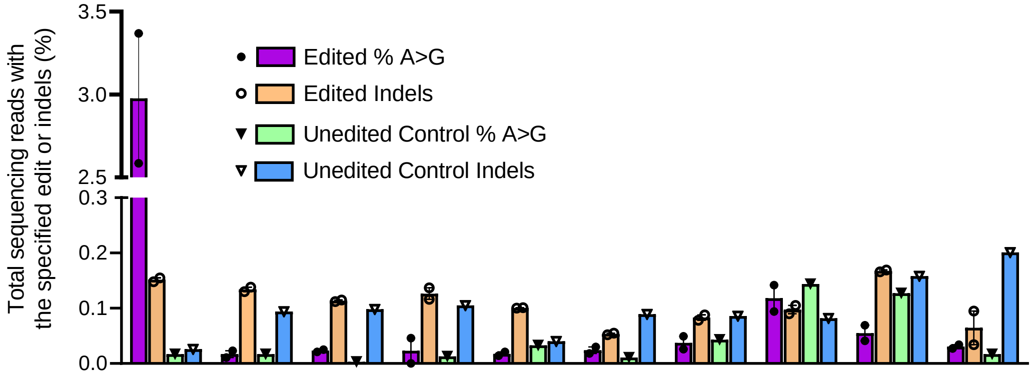

Supplementary Figure 2. Assessment of off-target base editing. Nine off-target sites identified by CIRCLE-seq and the target *CCR5* locus were amplified from genomic DNA extracted from epithelial cell brushings in two rhesus monkeys treated with S315 + ABE8e targeted to the *CCR5* test locus (Edited, closed and open circle symbols) and one negative control rhesus monkey treated with S315 and Cy5 (Unedited Control, closed and open triangle symbols). Base editing frequencies at position 4 of the aligned protospacer, as well as indel frequencies, were quantified using CRISPResso2 and plotted. Where applicable, the columns represent the averages of two replicate edited samples + SD, and the respective numerical values of individual data points are presented in Supplementary Table 6. Source data are provided as a Source Data File.

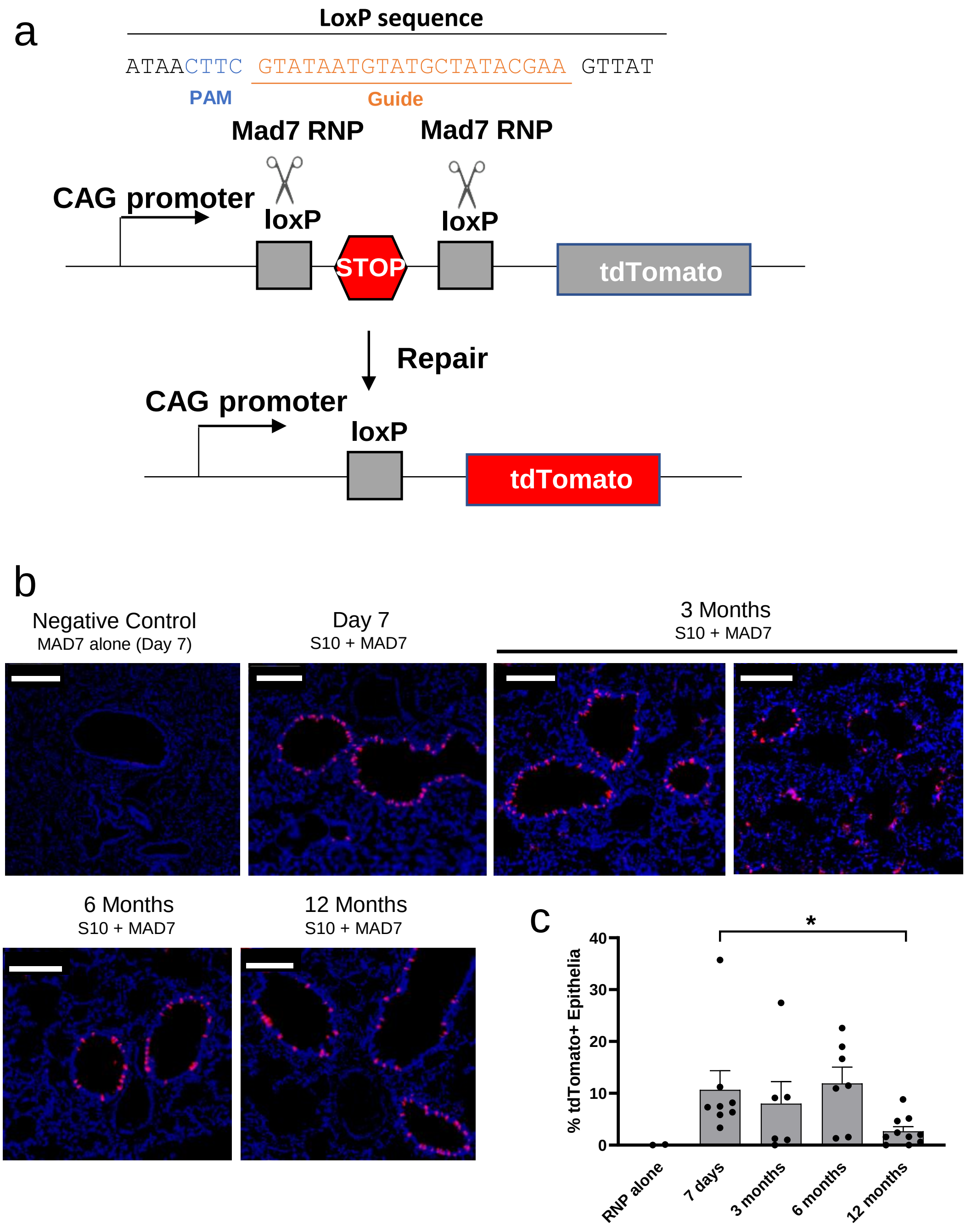
a
LoxP sequence
ATAACTTC GTATAATGTATGCTATACGAA GTTAT
PAM
Guide
Mad7 RNP
Mad7 RNP
CAG promoter
loxP
loxP
STOP
tdTomato
Repair
CAG promoter
loxP
tdTomato
b
Negative Control
MAD7 alone (Day 7)
Day 7
S10 + MAD7
3 Months
S10 + MAD7
6 Months
S10 + MAD7
12 Months
S10 + MAD7
c
*
% tdTomato+ Epithelia
40
30
20
10
0
RNP alone
7 days
3 months
6 months
12 months

Supplementary Figure 3. Persistence of gene editing in Ai9 mice following S10-mediated delivery of MAD7 nuclease. **a)** Schematic of reporter in *Rosa26* locus of Ai9 mice. PAM and gRNA to target LoxP sites are shown. **b)** Ai9 mice received MAD7 RNP with the S10 shuttle as described in the Methods section. At the indicated intervals, editing was assessed in lung tissue sections using fluorescence microscopy. Nuclease dependent editing is signified by tdTomato expression. Two images are shown at 3 months post-delivery to demonstrate editing within the airways and alveolar compartments. White scale bar indicates 200 µm. **c)** Quantification of tdTomato expression in airway epithelia at the indicated intervals. Each dot represents the count of an individual mouse and columns represent mean + SEM, P=0.023 by Kruskal-Wallis test. Source data are provided as a Source Data File.

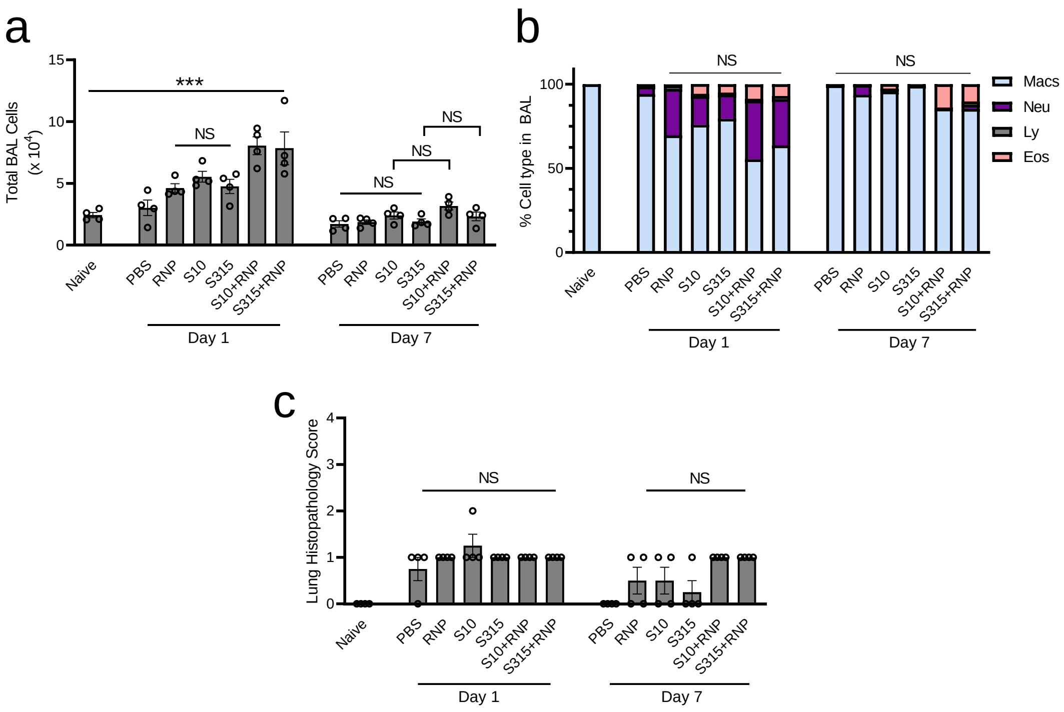

Supplementary Figure 4. Pulmonary toxicity study in mice following an intra-nasal delivery of the instillation solution components individually or in combination. **a)** Total cell counts in the BAL fluid on day 1 or 7 after the delivery, n=4 mice per group. Statistical analysis by one-way ANOVA (horizontal bars), ***$p<0.0001$, NS = non-significant; or two-sided t-test (brackets), NS = non-significant. **b)** Percentual proportion of the four cell types in the BAL fluid. Macs = macrophages (blue), Neu = neutrophils (purple), Ly = lymphocytes (gray), Eos = eosinophils (pink), n=4 mice per group. Statistical analysis by one-way ANOVA testing for the differences in % Neu between the groups. **c)** Degree of lung inflammation expressed as Lung Histopathology Score on the scale from 0 – 4 as described in the Methods, n = 4 mice per group. Statistics by one-way ANOVA. NS = non-significant. Source data are provided as a Source Data File.

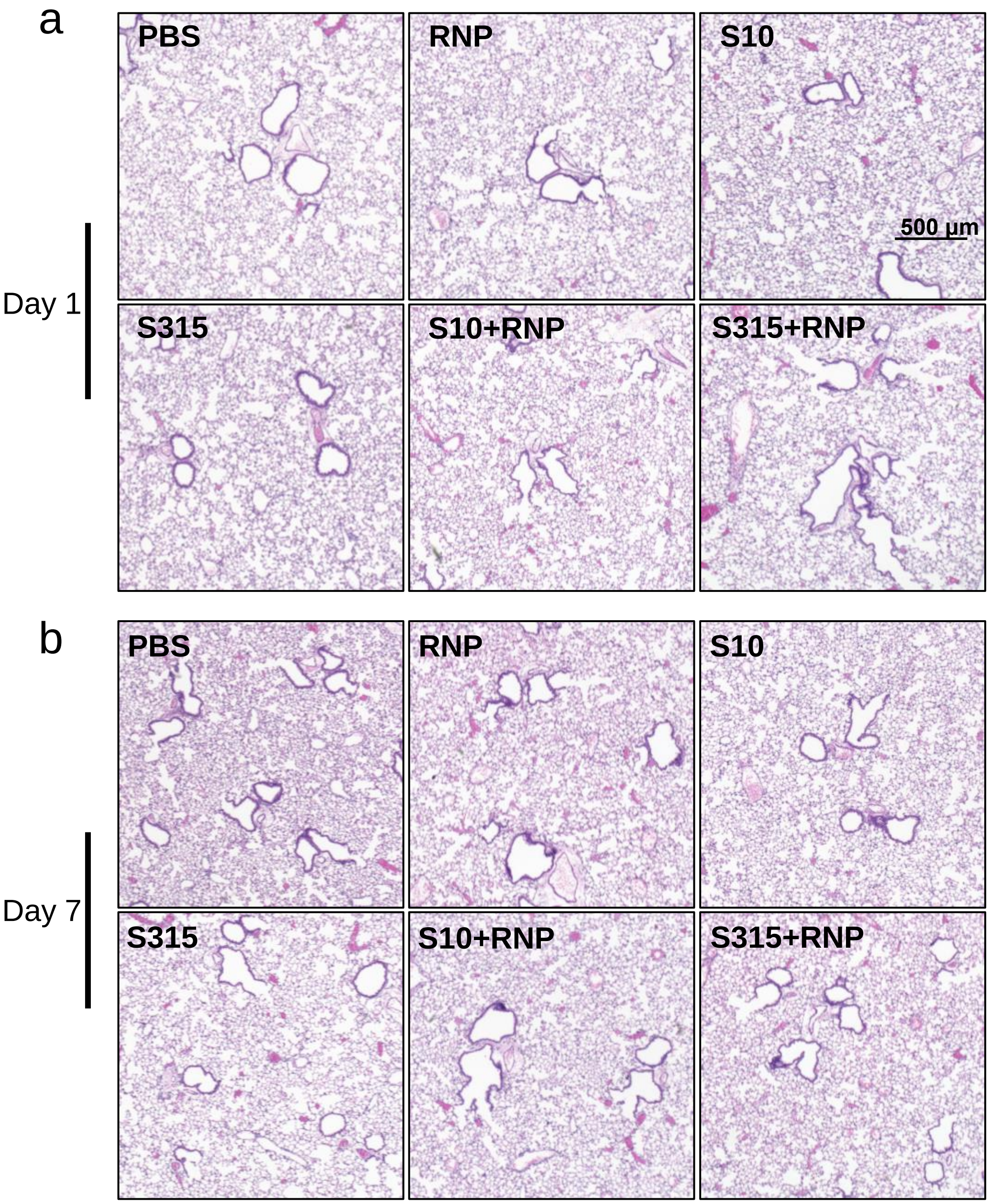

Supplementary Figure 5. Representative histopathology images of the mice' lungs after intra-nasal delivery of the gene editing components. Mice were instilled two times on two consecutive days with the indicated components (inset) and collected on Day 1 **(a)** or Day 7 **(b)** after the last instillation. Whole lung tissue sections stained with H&E were examined for inflammatory changes that were quantitatively analyzed and presented in Supplementary Figure 4c. N=4 mice per group. Scale bar 500 µm.

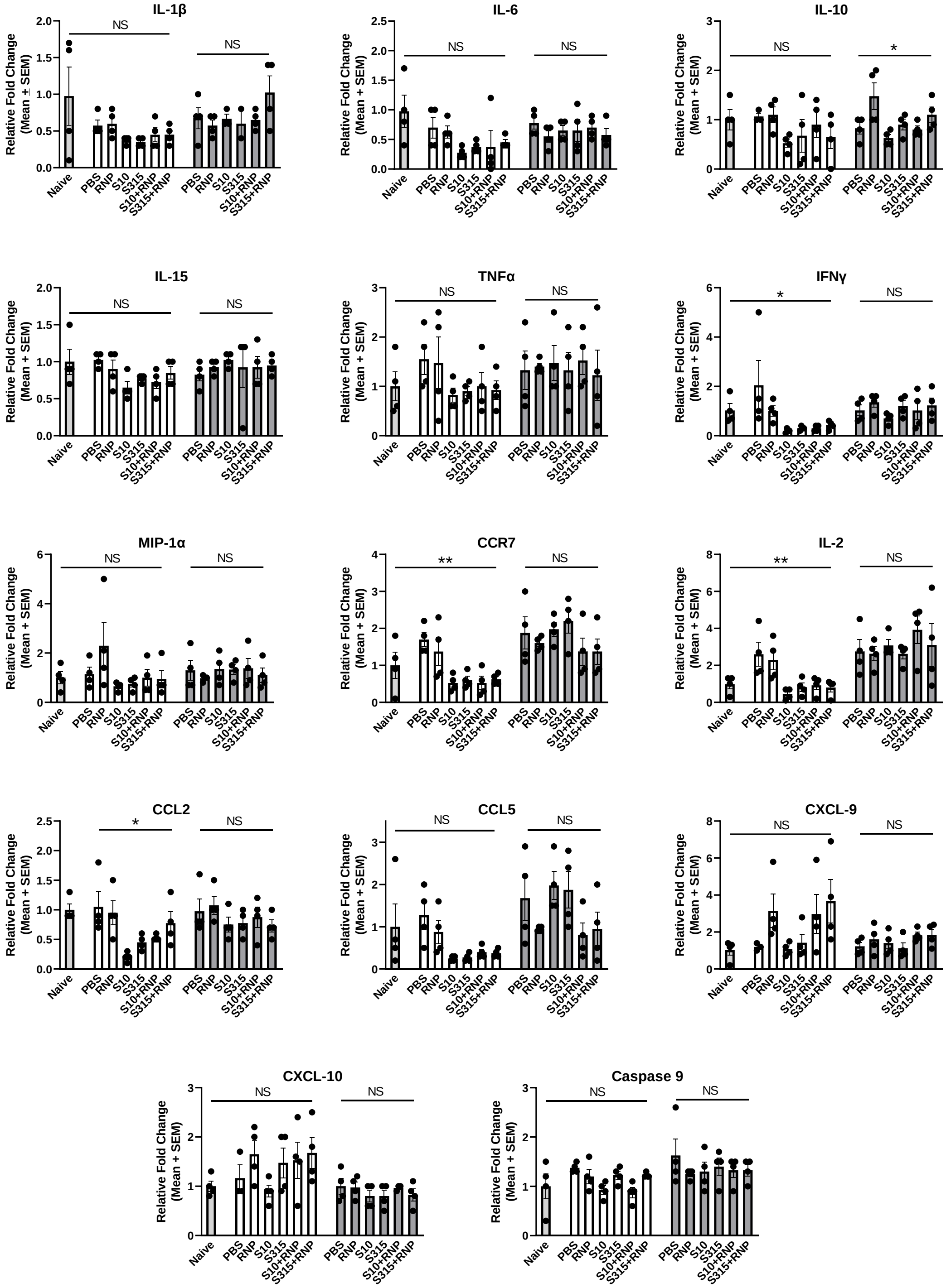
IL-1β
Relative Fold Change (Mean ± SEM)
NS
NS
Naive
PBS
RNP
S10
S315
S10+RNP
S315+RNP
IL-6
Relative Fold Change (Mean + SEM)
NS
NS
IL-10
NS
*
IL-15
NS
NS
TNFα
NS
NS
IFNγ
*
NS
MIP-1α
NS
NS
CCR7
**
NS
IL-2
**
NS
CCL2
*
NS
CCL5
NS
NS
CXCL-9
NS
NS
CXCL-10
NS
NS
Caspase 9
NS
NS

Supplementary Figure. 6. Pulmonary chemokine, cytokine, and protease mRNA transcript profile following the instillation of the gene editing solution components. Mice received two intra-nasal doses of the reagents indicated on the X-axis, lungs were collected on day 1 (white bars) and day 7 (dark gray bars) post instillation as described in the Methods, and the mRNA levels of the mediators in the whole lung homogenates were analyzed by RT qPCR. Data are expressed as a fold-change in mRNA expression relative to the Naïve group. Each symbol represents an individual mouse, and the height of the columns represents a mean +/- SEM. N=4 mice per group; significant outlier values identified by the Grubbs' test were removed (n=6 values). Statistics by one-way ANOVA, NS = non-significant; for IL-10 * p=0.014; for IFNγ * p=0.039; for CCR7 ** p=0.001, for IL-2 ** p=0.004; and for CCL2 * p=0.010. Source data are provided as a Source Data File.

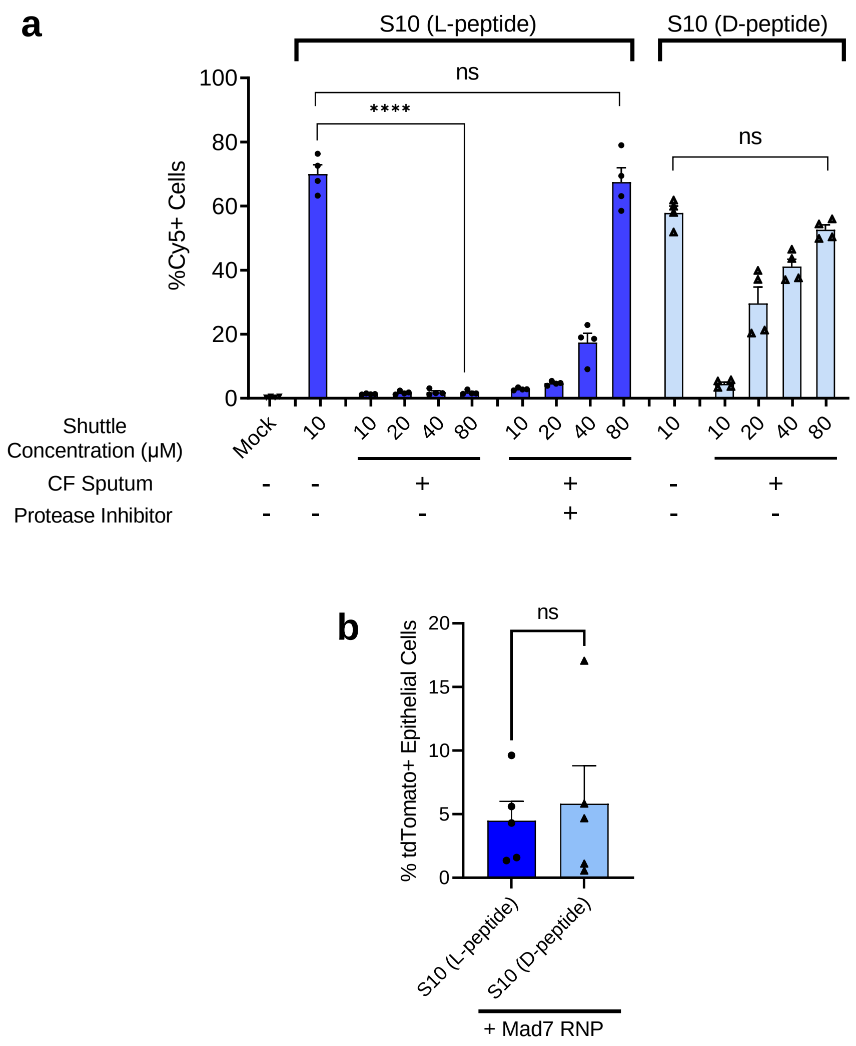

Supplementary Figure 7. D-peptide shuttle retains activity in the presence of CF sputum. a) Delivery of DRI-NLS-Cy5 to HeLa cells using L- or D-peptide S10 in the presence or absence of 10% CF sputum and/or protease inhibitor cocktail. Each closed circle or triangle represents a replicate from 2 CF sputum donors. **** denotes P=3.6E-07; ns: non-significant by two-tailed unpaired t-test (brackets). **b)** Quantification of tdTomato expression in Ai9 mouse airway epithelia 7 days post-delivery of Mad7 RNP using indicated shuttle peptide. Each dot represents the counting of an individual mouse and columns represent ±SEM. ns: non-significant by two-tailed unpaired t-test (brackets). Source data are provided as a Source Data File.

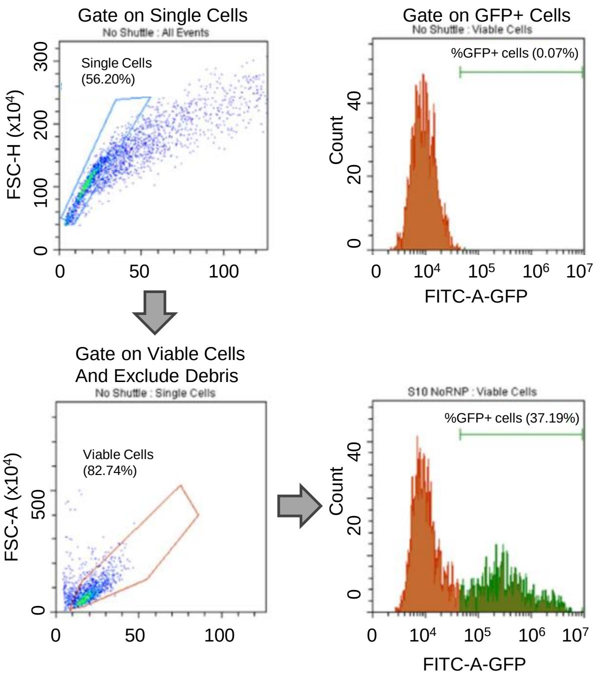

Tube name: No Shuttle
Volume (ul): 34.3

Population	Events	% Total	% Parent	Mean FITC-A
All Events	3000	100.00%	100.00%	29449.2
Single Events	1686	56.20%	56.20%	10558.6
Viable Cells	1395	46.50%	82.74%	10460.9
% GFP+ Cells	1	0.03%	0.07%	54633.3

Tube name: S10 NoRNP
Volume (ul): 35

Population	Events	% Total	% Parent	Mean FITC-A
All Events	3000	100.00%	100.00%	625834.4
Single Events	1676	55.87%	55.87%	232486.3
Viable Cells	1409	46.97%	84.07%	248077.8
% GFP+ Cells	524	17.47%	37.19%	630077.2

Supplementary Figure 8. Example of the flow cytometry gating strategy, related to data presented in **Fig. 1c, Suppl. Fig. 1.** 20,000 cells were seeded per condition. 3,000 events were recorded by flow cytometry per sample. Recorded cells were first gated on single cells (FSC-H/FSC-A) followed by gating to remove cell debris (SSC-A/FSC-A). The remaining cells were measured by respective fluorescence (FITC-A for GFP, APC-A for Cy5). Data plotted in Fig. 1c and Suppl. Fig 1 represent the results presented in the FITC-A-GFP FACS data panel for GFP+ cells, and alternatively APC-A-Cy5 data panel for Cy5+ cells (not shown).

Peptide ID	Amino Acid Sequence	Mean % Editing
S199	LARAFARALLKLWGQRRLKAKRA	1.98%
S114	LARAFARAIKIFGQRRLKAKRA	2.99%
S318	KLKLAKALAKALKKLGGSGGGSKKLKAKKA	3.14%
S301	KLKLAKALAKALKKLGGSGGGSQAKALAKQAK	3.29%
S115	KWKLARAFARAIKIFGGSGGGSQRRLKAKRA	3.55%
S10	KWKLARAFARAIKKLGGSGGGSYARALRRQARTG	3.88%
S321	KWKLAKAFAKAIKKLGGSGGGSYAKALKKQAKTG	7.14%
S195	LARAFARAIKIFGQARAQARQAR	8.34%
S262	LAKALAKALKLLGQARAQARQAR	9.86%
S315	KLKLAKALAKALKLLGGSGGGSQAKAQAKQAK	11.20%

Supplementary Table 1. Amino acid sequences of the delivery peptides screened in Figure 1a and their associated mean editing efficiency in the human airway epithelial cells cultured at the air liquid interface.

Group/ Main Experimental Goal	Animal Number	aCargo (Concentration)	Shuttle Peptide (Concentration)	Tissues Harvest Time post Delivery	Main Tissues Collected
Group 1/ Biodistribution	1	DRI-NLS-Cy5 (20 µM)	-	1 hr	Lungs fixed in 4%PF, one lobe fresh tissue for brushing
	2	DRI-NLS-Cy5 (20 µM)	S10 (40 µM)	1 hr	Lungs fixed in 4% PF, one lobe fresh tissue for brushing
	3	DRI-NLS-Cy5 (10 µM)	-	2 hrs	Lungs fixed in 4% PF, one lobe fresh tissue for brushing
	4	DRI-NLS-Cy5 (10 µM)	S10 (40 µM)	2 hrs	Lungs fixed in 4% PF, one lobe fresh tissue for brushing
Group 2/ CCR5 Gene Editing	5	RNP: ABE8e-Cas9/gRNA duplex (2.5 µM/2 µM)	-	7 days	Airway brushings
	6	RNP: ABE8e-Cas9/gRNA duplex (2.5 µM/2 µM)	S10 (40 µM)	7 days	Airway brushings
	7	RNP: ABE8e-Cas9/gRNA duplex (2.5 µM/2 µM)	S315 (40 µM)	7 days	Airway brushings
	8	RNP: ABE8e-Cas9/gRNA duplex (2.5 µM/2 µM)	S315 (40 µM)	7 days	Airway brushings

Supplementary Table 2. Experimental rhesus monkey groups and the interventions they received. Treatment groups specifying the instillation solutions components and concentrations, time of tissues harvest, and main lung tissue processing.

Target Gene	gRNA sequence	PAM	Forward Primer*	Reverse Primer*
CFTR	CTTGATTTCTGGAGACCACA	AGG	CTCTCAAAATGCCTACTGGGAAC	GGCTAGAGTACTTCCCGCA
CFTR R553X	TTGCTCATTGACCTCCACTC	AG	GGAAGATGTGCCTTTCAAATTCAG	ATGTGATTCTTAACCCACTAGCCA
B2M	GAGTAGCGCGAGCACAGCTA	AGG	CTGGGCACGCGTTTAATATAAG	CACCAAGGAGAACTTGGAGAA
CCR5	GAGAGTTTCTTGTAGGGGAA	CGG	ACTGTCTATATGATTGATTTGCACAAC	CCTTTAAAGTCTTTCACTCACAATC

Supplementary Table 3. Sequences of gRNAs and primers used. Adapters were added to the primer in the following format. Forward Primer Sequence: ACACTCTTTCCCTACACGACGCTCTTCCGATCTNNNN. Reverse Primer Sequence: TGGAGTTCAGACGTGTGCTCTTCCGATCT

Site Name	Site Sequence w/mismatch or gap	PAM	Chromosome	Position	Strand	Mismatch
CCR5 Control	GAGAGTTTCTTGTAGGGGAA	CGG	2	102388761	-	0
OT1	cAGAGTTTCTT-TAGGaaAA	NGG	6	51031143	+	3 + 1 nt gap
OT2	TgGAGTcTCTTGTAGGGGAA	NGT	20	13884468	-	3
OT3	GAGAGaTatTTGTAGGGGAg	NGA	7	44406298	+	4
OT4	GAGAGTTTCTTtTAGGaGAt	NGG	4	9686016	-	3
OT5	aAGAaTTTCTTGTAGGt-AA	NGG	3	115726304	+	3 + 1 nt gap
OT6	GAGAGTgaCTTGTAGGGGt-	NGG	12	6412404	-	3 + 1 nt gap
OT7	ctGAGTTTCTTG-AGGGaAA	NGG	8	125235221	+	3 + 1 nt gap
OT8	GAGAGcTcCTTaaAGGGaAA	NGG	4	140936131	+	5
OT9	GAGAGTTTCTTa-AGaGGAA	NGG	9	114099813	-	2 + 1 nt gap
OT10*	AAGAGTTTCTTGTgGGGGAg	NGG	4	62577684	-	3
OT11*	CAGAGTcTCTTGcAGGGaAt	NAG	20	3060622	+	5
OT12*	aAGAGTTaCTTG-AGGGtAA	NGG	7	128513075	-	3 + 1 nt gap
OT13*	AAGAGcTTCTTGcAGGGGAA	NGA	2	70230618	+	3
OT14*	CAGAGTTTCTTGTAGGGGgg	NTG	13	793132	-	3

Supplementary Table 4. Off-target sites of rhesus monkey CCR5-gRNA identified by CIRCLE-seq. Mismatches are denoted by lower case letters, and gaps by a hyphen. * denotes sites that did not amplify.

Off-Target	Site Sequence	Forward Primer*	Reverse Primer*
OT1	CAGAGTTTCTTTAGGAAAAGGG	ACTGATCAGGCAGGGTGACAAT	GATGCACGGCTTTGAGGAAAAACC
OT2	TGGAGTCTCTTGTAGGGGAAGGT	TTGGTTCCCAGGCTTACCTGG	TGGGTGTAGAAGAGAGGAGGGGA
OT3	GAGAGATATTTGTAGGGGAGGGA	CCAGGGAAGCTCTGTAGAAGAG	CCAATCCATCTTCCACAGCTC
OT4	GAGAGTTTCTTTTAGGAGATGGG	CACACGCCTTTCACCACATCAC	ACTCAGCATCTCAATGGAGGG
OT5	AAGAATTTCTTGTAGGTAAAGGA	TGGCTCTCCCAGCAATTTGA	GGAACTGTAGAAACTCTCCTCGAG
OT6	GAGAGTGACTTGTAGGGGTGGGT	ACTGAATTCCCGGCCATGAC	GCAATGGGTTAGAGGAGGAAAGC
OT7	CTGAGTTTCTTGAGGGAAAGGG	GACAACTTCAGTCAAATCAGCAGAC	ATTTCGTCCTGGGTACCTGG
OT8	GAGAGCTCCTTAAAGGGAAAGGG	GGGGATAAGCAAGACAGCTATC	CCAGTGGATGGATGGAACTGG
OT9	GAGAGTTTCTTAAGAGGAAGGG	CTCTGCTTTAAGCAGGCACCA	CAAGAGCATTCCTCTGTGGCC
OT10	AAGAGTTTCTTGTGGGGGAGGGG	GAATTCAGCAACCACTGAGCCA	GCAGTTGGAATAAACTGGCACGC
OT11	CAGAGTCTCTTGCAGGGAATGAG	CCTCTTTCCTCCTCCAGAGTTCC	TGTGGGGAGGAAAGCTGGCA
OT12	AAGAGTTACTTGAGGGTAAAGG	CAAACATTGCAGAACCTGAAAGTAC	TGATCATCCTCAGTGATTCTGGTTC
OT13	AAGAGCTTCTTGCAGGGGAAGGA	GGCTAATTTGGGCCATATATG	GCAATTCTCAGGACCCATTT
OT14	CAGAGTTTCTTGTAGGGGGGATG	TTCGTAGAGATGGGGTGTCACC	GCTAAGTGACAGAAGCCAGCC

Supplementary Table 5. DNA region sequences amplified for off-target analysis and primers used. Adapters were added to the primer in the following format: Forward Primer Sequence: ACACTCTTTCCCTACACGACGCTCTTCCGATCTNNNN. Reverse Primer Sequence: TGGAGTTCAGACGTGTGCTCTTCCGATCT.

DNA Region	Unedited Control % A>G	Unedited Control Indels	Edited % A>G*	Edited Indels*
CCR5	0.017	0.026	2.977	0.151
OT1	0.017	0.094	0.017	0.134
OT2	0.003	0.098	0.023	0.114
OT3	0.013	0.105	0.023	0.126
OT4	0.033	0.040	0.017	0.100
OT5	0.011	0.089	0.024	0.054
OT6	0.043	0.086	0.038	0.083
OT7	0.144	0.082	0.118	0.097
OT8	0.127	0.158	0.055	0.168
OT9	0.017	0.201	0.031	0.065

Supplementary Table 6. Assessment of off-target base editing - values corresponding to the Supplementary Fig 2. * Average of 2 replicate edited samples from different rhesus monkeys.
