# Supplementary material for: Shuttle peptide delivers base editor RNPs to rhesus monkey airway epithelial cells in vivo
Source: Nat Commun. 2023 Dec 5;14:8051. doi: 10.1038/s41467-023-43904-w (PMC10698009; doi:10.1038/s41467-023-43904-w)
Supplement: Supplementary file 3 — Reporting Summary [file 41467_2023_43904_MOESM3_ESM.pdf]

nature portfolio

Corresponding author(s): Paul B. McCray

Last updated by author(s): Oct 23, 2023

Reporting Summary

Nature Portfolio wishes to improve the reproducibility of the work that we publish. This form provides structure for consistency and transparency in reporting. For further information on Nature Portfolio policies, see our Editorial Policies and the Editorial Policy Checklist.

Statistics

For all statistical analyses, confirm that the following items are present in the figure legend, table legend, main text, or Methods section.

n/a	Confirmed	
☐	☒	The exact sample size (*n*) for each experimental group/condition, given as a discrete number and unit of measurement
☐	☒	A statement on whether measurements were taken from distinct samples or whether the same sample was measured repeatedly
☐	☒	The statistical test(s) used AND whether they are one- or two-sided<br>*Only common tests should be described solely by name; describe more complex techniques in the Methods section.*
☒	☐	A description of all covariates tested
☐	☒	A description of any assumptions or corrections, such as tests of normality and adjustment for multiple comparisons
☐	☒	A full description of the statistical parameters including central tendency (e.g. means) or other basic estimates (e.g. regression coefficient) AND variation (e.g. standard deviation) or associated estimates of uncertainty (e.g. confidence intervals)
☐	☒	For null hypothesis testing, the test statistic (e.g. *F*, *t*, *r*) with confidence intervals, effect sizes, degrees of freedom and *P* value noted<br>*Give P values as exact values whenever suitable.*
☒	☐	For Bayesian analysis, information on the choice of priors and Markov chain Monte Carlo settings
☒	☐	For hierarchical and complex designs, identification of the appropriate level for tests and full reporting of outcomes
☒	☐	Estimates of effect sizes (e.g. Cohen's *d*, Pearson's *r*), indicating how they were calculated

Our web collection on statistics for biologists contains articles on many of the points above.

Software and code

Policy information about availability of computer code

Data collection	https://github.com/pinellolab/CRISPResso2<br>https://github.com/tsailabSJ/circleseq
Data analysis	GraphPad Prism 9, SAS v 9.4

For manuscripts utilizing custom algorithms or software that are central to the research but not yet described in published literature, software must be made available to editors and reviewers. We strongly encourage code deposition in a community repository (e.g. GitHub). See the Nature Portfolio guidelines for submitting code & software for further information.

Data

Policy information about availability of data

All manuscripts must include a data availability statement. This statement should provide the following information, where applicable:

- Accession codes, unique identifiers, or web links for publicly available datasets
- A description of any restrictions on data availability
- For clinical datasets or third party data, please ensure that the statement adheres to our policy

All relevant data supporting the key findings of this study and any associated accession codes and references are available within the article and in the Supplementary Information files or from the corresponding authors upon request. Source data for all figures are provided with the paper. High-throughput sequencing data have been deposited in the NCBI Sequence Read Archive database under accession code [to be supplied later].

Research involving human participants, their data, or biological material

Policy information about studies with human participants or human data. See also policy information about sex, gender (identity/presentation), and sexual orientation and race, ethnicity and racism.

Reporting on sex and gender	Sputum samples from anonymous CF patients were obtained from the Primary Airway Cell Biobank (PACB) of McGill University, in Montréal, Canada. No data on sex/gender is available.
Reporting on race, ethnicity, or other socially relevant groupings	No data on race, ethnicity, or other socially relevant groupings is available.
Population characteristics	No data on population characteristics is available.
Recruitment	No data on recruitment is available.
Ethics oversight	Sputum collection followed strict ethical guidelines and patient anonymity was preserved. The study was approved by the IRB of McGill University (IRB # A08-M70-14B).

Note that full information on the approval of the study protocol must also be provided in the manuscript.

Field-specific reporting

Please select the one below that is the best fit for your research. If you are not sure, read the appropriate sections before making your selection.

☒ Life sciences ☐ Behavioural & social sciences ☐ Ecological, evolutionary & environmental sciences

For a reference copy of the document with all sections, see nature.com/documents/nr-reporting-summary-flat.pdf

Life sciences study design

All studies must disclose on these points even when the disclosure is negative.

Sample size	Rhesus monkey study used a minimum n=1 and maximum n=2 animals per treatment group, the minimum necessary to obtain valid and meaningful results, and document reproducibility in non-human primate model. Mice studies used between n = 4 - 10 animals per treatment group, with a control group that was expected not to differ from a not treated animals consisting of n = 2. These numbers were selected for both the short, and the long-term 12-month study to reliably detect the expected effect size without unnecessary repetition of the study. In vitro studies used between 3 - 6 cultures per treatment group. These sample sizes were determined as the minimum to detect differences between the treatments at the minimum level of significance of $p<0.05$ and power 80%.
Data exclusions	6 out of 728 data points in the Fig S6 (cytokine levels by RT qPCR) were excluded from analysis as significant outliers based on Grubbs' test. We used this test to determine whether the most extreme value in a group is a significant outlier from the rest. The Grubbs' test utilizes an ESD method (extreme studentized deviate). No other data were excluded from analysis.
Replication	To verify the reproducibility of the experimental findings we utilized both technical and biological replicates, and/or performed 2 or more independent experiments. The replication requirement in rhesus monkey experiments was addressed by using a biological replicate – two monkeys that received the identical treatment and exhibited concordant results. Where applicable, the experiments were performed two or more times and yielded similar results.
Randomization	Individual animals and in vitro samples from human donors were randomly allocated into experimental groups.
Blinding	The CT scans obtained before and after treatment of the rhesus monkeys were scored for areas of ground glass changes or consolidation post-procedure by a pulmonologist blinded to the experimental conditions. H&E stained lung tissues from mice were examined by a board certified veterinary pathologist using the post-examination method of masking group assignment. The other analysis did not require blinding as the type and magnitude of the responses were unequivocal between the treatment and control groups.

Reporting for specific materials, systems and methods

We require information from authors about some types of materials, experimental systems and methods used in many studies. Here, indicate whether each material, system or method listed is relevant to your study. If you are not sure if a list item applies to your research, read the appropriate section before selecting a response.

Materials & experimental systems

n/a	Involved in the study	
☐	☒	Antibodies
☐	☒	Eukaryotic cell lines
☒	☐	Palaeontology and archaeology
☐	☒	Animals and other organisms
☒	☐	Clinical data
☒	☐	Dual use research of concern
☒	☐	Plants

Methods

n/a	Involved in the study	
☒	☐	ChIP-seq
☐	☒	Flow cytometry
☒	☐	MRI-based neuroimaging

Antibodies

Antibodies used

The following staining antibodies were used: Keratin 5 Polyclonal Antibody to stain basal cells (CK5, BioLegend, Ref 905501, Clone Poly19055), SFTPC Polyclonal Antibody to stain surfactant protein C-secreting cells (Invitrogen, Ref PA5-71680), Anti-Club Cell Secretory Protein Antibody to stain secretory cells (SCGB1A1, Millipore, Ref 07-623, polyclonal), and Monoclonal Anti-Tubulin Acetylated Antibody to stain the ciliated cells (AcTub, Sigma-Aldrich, Ref T6793, clone clone 6-11B-1). Primary antibodies were used at concentrations at 1:50 – 1:100, and secondary antibodies at 1:500 – 1:1,000.

Validation

The Sigma-Aldrich lists the T6793 antibody reactive to monkey, among other species. To validate the other antibodies we utilized a combination of the following: if antibodies are reactive to human, we considered a close homology between non-human primates and human; detailed analysis of morphological features of the staining pattern; and previous experience using the antibodies. Antibody T6793 is a monoclonal anti-tubulin, acetylated, antibody provided by Sigma-Aldrich. It is a clone 6-11B-1, raised in mouse, with a reported species reactivity to human, monkey,
mouse, protista, invertebrates, hamster, plant, frog, rat, pig, bovine, and chicken.

According to the manufacturer, "the antibody has been used to detect acetylated α-tubulins from many organisms that are frequently studied in the laboratory: protista, plants, invertebrates and vertebrates (e.g., human, mouse, pig, bovine, rat, hamster, monkey, chicken, frog). Details on the strains
of organisms and microtubule structures containing acetylated α-tubulin detected by the antibody have been described" by LeDizet et al.

LeDizet, M., and Piperno, G., Meth. Enzymol., 196, 264 (1991). Detection of acetylated α-tubulin by specific antibodies; https://doi.org/10.1016/0076-6879(91)96025-M

According to Labome, the Validated Antibody Database, (https://www.labome.com/review/gene/human/alpha-tubulin-antibody.html) there is 1,750 published articles using this antibody.
Among those many use them for various applications on human samples/cells. The T6793 antibody was used in western blot on human samples (Fig 1) and in immunocytochemistry on African green monkey samples (Fig 6). J Pharmacol Exp Ther (2016) https://pubmed.ncbi.nlm.nih.gov/26980057/

Antibody data sheet: https://www.sigmaaldrich.com/deepweb/assets/sigmaaldrich/product/documents/406/554/t6793dat.pdf

UniProt accession no: https://www.uniprot.org/uniprotkb/P68368/entry

CiteAb entry: https://www.citeab.com/antibodies/2304915-t6793-monoclonal-anti-tubulin-acetylated-antibody-p

Eukaryotic cell lines

Policy information about cell lines and Sex and Gender in Research

Cell line source(s)

CFF-16HBEge CFTR R553X cell line was kindly provided by the Cystic Fibrosis Foundation, and subsequently banked and cultured at the Feldan Therapeutics. The CFF-16HBEge R553X was developed from the primary cells isolated from a male. HeLa cell line (developed from cells isolated from a female) were used in some experiments. The HeLa cells were obtained from and validated by ATCC (CCL-2).

Authentication

CFF-16HBEge CFTR R553X cell line was obtained from and authenticated by the Cystic Fibrosis Foundation. Repeated authentication in the laboratory was not performed. Similarly, the HeLa cells were obtained from and validated by ATCC (CCL-2), and an additional authentication in the laboratory was not performed.

Mycoplasma contamination

The regular mycoplasma testings at both the Feldan Therapeutics and the University of Iowa documented the negativity of the tested cell cultures, but the specific cell lines and/or cells used in this study (CFF-16HBEge CFTR R553X, HeLa) were not tested.

Commonly misidentified lines
(See ICLAC register)

No misidentified cell lines were used.

Animals and other research organisms

Policy information about studies involving animals; ARRIVE guidelines recommended for reporting animal research, and Sex and Gender in Research

Laboratory animals	The following two strains of mice were used:<br>1) Ai9 (B6.Cg-Gt(ROSA)26Sortm9(CAG-tdTomato)Hze/J mice (from Jackson Labs, Strain #007909; on a genetic background Strain #000664 C57BL/6J).<br>2) C57BL6/J mice (Jackson Labs, Strain # 000664)<br>Mice between ages 6-10 weeks of both sexes were randomly assigned to treatment groups. Information on the housing conditions were provided in the manuscript.<br><br>The Rhesus macaques used in the study were of both genders, about 4-5 months old.
Wild animals	The study did not involve wild animals.
Reporting on sex	Animals of both sexes were used in all the animal experiments, and no sex-based analyses were performed.
Field-collected samples	The study did not involve samples collected from the field.
Ethics oversight	For the Rhesus monkeys studies, all procedures conformed to the requirements of the Animal Welfare Act, and protocols were approved prior to implementation by the Institutional Animal Care and Use Committee (IACUC) at the University of California, Davis.<br>The mice studies at TransBIOtech were performed according to the institutional guidelines approved by the CEGEP de Lévis Animal Care Committee (Lévis, Quebec, CA; Approval 024-20), and complied with CACC standards and regulations governing the use of animals for research.<br>The mice studies at the University of Iowa were performed in accordance with the University of Iowa's Institutional Animal Care and Use Committee (IACUC, Animal Protocol # 7072030-008) and in accordance with National Institutes of Health guidelines.

Note that full information on the approval of the study protocol must also be provided in the manuscript.

Plants

Seed stocks	No seed stocks were used in this study.
Novel plant genotypes	No novel plant genotypes were used in this study.
Authentication	N/A

Flow Cytometry

Plots

Confirm that:

- ☒ The axis labels state the marker and fluorochrome used (e.g. CD4-FITC).
- ☒ The axis scales are clearly visible. Include numbers along axes only for bottom left plot of group (a 'group' is an analysis of identical markers).
- ☒ All plots are contour plots with outliers or pseudocolor plots.
- ☒ A numerical value for number of cells or percentage (with statistics) is provided.

Methodology

Sample preparation	CFF-16HBEge CFTR R553X cell line was kindly provided by Cystic Fibrosis Foundation. 20,000 cells were seeded per well in 96-well plate in alpha-MEM medium supplemented with 10% FBS and 1% of Penicillin-Streptomycin-Glutamine the day before the experiment. After delivery, cells were dissociated using the TripLE for 20min at 37 degrees C before analyzed by flow cytometry.
Instrument	CytoFLEX (Serial number: BB26201), Beckman Coulter.
Software	CytExpert for the CytoFLEX platform (Beckman Coulter Life Sciences)
Cell population abundance	20,000 cells were seeded per condition. 3,000 events were recorded by flow cytometry per sample. Between ~1,400-1,670

Cell population abundance	events remained before the final gating on GFP+ or Cy5+ cells. The abundance and percentage of the final GFP+ or Cy5+ events were gated based on FITC-A (area) or APC-A filter parameter, respectively.
Gating strategy	1. Gate on single cell events (FSC-A/FSC-H); 2. Eliminate debris (FSC-A/SSC-A); 3. Gate on GFP positive cells (FITC-A level) or Cy5+ (APC-A). The boundaries between the GFP or Cy5 negative and positive populations were set based on the control sample.

☒ Tick this box to confirm that a figure exemplifying the gating strategy is provided in the Supplementary Information.
